# Draft Genome Sequence of *Pseudomonas aeruginosa* Strain RB, a Bacterium Capable of Synthesizing Cadmium Selenide Nanoparticles

**DOI:** 10.1128/genomeA.00368-14

**Published:** 2014-05-15

**Authors:** Hiroyuki Ayano, Masashi Kuroda, Satoshi Soda, Michihiko Ike

**Affiliations:** Division of Sustainable Energy and Environmental Engineering, Graduate School of Engineering, Osaka University, Yamadaoka, Suita, Osaka, Japan

## Abstract

*Pseudomonas aeruginosa* strain RB is a bacterium capable of synthesizing cadmium selenide (CdSe) nanoparticles and was isolated from a soil sample. Here, we present the draft genome sequence of *P. aeruginosa* strain RB. To the best of our knowledge, this is the first report of a draft genome of a CdSe-synthesizing bacterium.

## GENOME ANNOUNCEMENT

Quantum dots (QDs) are 1- to 10-nm semiconductor nanoparticles that possess size-dependent luminescence (1). Cadmium selenide (CdSe) QDs are used in light-emitting diodes, solar cells, and biological imaging (2). Recently, the microbial synthesis of CdSe at ambient temperature and pressure without toxic solvents has garnered considerable attention as an environmentally friendly procedure (3).

The bacterial RB strain was isolated from a soil sample as a CdSe nanoparticle-synthesizing bacterium (4). It has the capacity to reduce selenite to selenide, is cadmium resistant, and produces CdSe nanoparticles from selenite and cadmium ions in a one-vessel operation. The resultant CdSe nanoparticles accumulate inside and on the surface of the cells. Using homology searches, the genome of strain RB was revealed to be very similar to that of *Pseudomonas aeruginosa*. This is the first report of the genome sequence of a bacterium that synthesizes CdSe nanoparticles.

The genomic DNA of strain RB was fragmented and prepared into a sequence library according to the instructions of the TruSeq DNA sample preparation kit (Illumina, San Diego, CA, USA). Multiplex sequencing was performed using the HiSeq 2000 and 101-bp paired-end reads, resulting in 3.7 Gbp of reads. From the sequenced reads, the adapter sequences were trimmed using the cutadapt program (http://code.google.com/p/cutadapt). After the adapters were trimmed, the reads were assembled onto the draft genome sequence by using Velvet program version 1.2.08 (http://www.ebi.ac.uk/~zerbino/velvet/). The sequence contains 78 contigs, with a G+C content of 66.6%, accounting for a total of 6,200,537 bp, with an *N*_50_ of 712,616 bp and a maximum contig size of 898,733 bp. A total of 5,690 coding sequences (CDSs) were predicted by the Rapid Annotations using Subsystems Technology (RAST) pipeline (5) and were found to contain 1,080 hypothetical protein genes, 63 tRNAs, and 2 rRNAs.

Fourteen genes that encode enzymes related to selenocompound metabolism and a single gene encoding selenoprotein O were identified in the strain RB genome by using the Kyoto Encyclopedia of Genes and Genomes (KEGG) Pathway Database (6). Six genes encoding proteins that catalyze the uptake of selenate and selenite were also found in the RB strain genome. The RB strain genome contains 31 genes encoding proteins related to cobalt-zinc-cadmium resistance (*czc*) and a gene encoding metallothionein. In addition, genes encoding a zinc ABC transporter (*znuA*, *znuB*, and *znuC*) and a chromate transport protein (*chrA*), 5 genes encoding arsenical resistance proteins, and 23 genes encoding proteins involved in copper homeostasis, tolerance, or transport were also identified. A comparative analysis revealed that strain RB shares most of these genes that are related to selenium metabolism and heavy metal resistance with 9 other strains of *P. aeruginosa* (19BR, DK2, MPAO1/P2, NCGM2.S1, 2192, PAO1, UCBPP-PA14, LESB58, and M18). The numbers of these genes possessed by these strains differed slightly, suggesting that the capacity to synthesize CdSe nanoparticles might be a common ability of all *P. aeruginosa* strains. The CdSe-synthesizing mechanism used by strain RB should be elucidated using gene expression analyses.

### Nucleotide sequence accession numbers.

The draft genome sequence and annotation are accessible from the DDBJ database under accession no. BAUN01000001 to BAUN01000102 and DF396803 to DF396880, respectively.
